# The early in utero oestrogen and testosterone environment of blacks and whites: potential effects on male offspring.

**DOI:** 10.1038/bjc.1988.46

**Published:** 1988-02

**Authors:** B. E. Henderson, L. Bernstein, R. K. Ross, R. H. Depue, H. L. Judd

**Affiliations:** Department of Preventive Medicine, University of Southern California, School of Medicine, Los Angeles.


 © The Macmillan Press Ltd., 1988

SHORT COMMUNICATION

### The early *in utero* oestrogen and testosterone environment of blacks and whites: Potential effects on male offspring

B.E. Henderson[1], L. Bernstein[1], R.K. Ross[1], R.H. Depue[2]* & H.L. Judd[3]

[1]*Department of Preventive Medicine, University of Southern California, School of Medicine, Los Angeles, California;* [2]*Division of Cancer Etiology, National Cancer Institute, Bethesda, Maryland; and* [3]*Department of Obstetrics and Gynecology, University of California, School of Medicine, Los Angeles, California, USA*

The seminal discovery of Herbst and his coworkers (1971, 1986) that early *in utero* exposure to diethylstilboestrol (DES) is associated with adolescent and young adult adenocarcinoma of the vagina in female offspring led us to suggest a related mechanism for the development of germ cell tumours of the testis (Henderson *et al.*, 1983). These testis tumours occur most frequently in young adult males and the age-specific incidence curve shows a broad peak between 20 and 39 years of age (Ross *et al.*, 1979; Newell *et al.*, 1984). Several studies have examined the risk of testis cancer associated with *in utero* exposure to exogenous oestrogens. Two of these found few subjects with such exposures. In one there was no evidence of an increased risk (Brown *et al.*, 1986) and in the other there was a positive, but not statistically significant, association with DES only (Moss *et al.*, 1986). We and others observed a strong positive association between *in utero* exposure in early pregnancy to DES or other oestrogenic substances and risk of testis cancer in the offspring (Henderson *et al.*, 1979; Schottenfeld *et al.*, 1980; Depue *et al.*, 1983). Based on these positive findings, we formulated an hypothesis which stated that, in such cases, the risk of germ cell tumours of the testis was determined *in utero* by the abrupt change in oestrogen levels resulting from the administration of exogenous oestrogens which interrupted the progression of primitive germ cells to mature germ cells (Henderson *et al.*, 1982, 1983). These primitive germ cells, persisting into the pubertal period, would multiply under stimulation by gonadotrophins and give rise to germ cell tumours of a variety of histological types depending on their particular stage of 'developmental arrest'.

Some support for this hypothesis can be found in the experimental literature. Yasuda *et al.* (1985*a*) have reported the persistence of gonadocytes following *in utero* administration of oestrogen to mice. Testicular anomalies and testicular maldescent (i.e., cryptorchidism, a major risk factor for testis cancer) can be produced by the administration of DES to mice and rats (Burns, 1955; McLachlin *et al.*, 1975; Walker, 1980; Yasuda *et al.*, 1985*b*).

In further analyses of our two case-control studies of testis cancer, we also observed risk to be associated with maternal obesity prior to, and hyperemesis during, the index pregnancy (Henderson *et al.*, 1979; Depue *et al.*, 1983). Obesity may reflect higher endogenous oestrogen levels as it has been found to be associated with increased levels of oestradiol ($E_2$) in post-menopausal women, particularly the 'free' or non-protein bound fractions, which may be due, in part, to the related decreased levels of sex hormone binding globulin (Nisker *et al.*, 1980). Although nausea and vomiting of pregnancy have been ascribed by some to the high and rising human chorionic gonadotrophin (hCG) levels of the first trimester (Kauppila *et al.*, 1979), others have been unable to relate these conditions to higher hCG (Soules *et al.*, 1980). We recently reported that hyperemesis may be similarly associated with excess levels of free $E_2$ during the initial weeks of pregnancy (Depue *et al.*, 1987). These two observations have led us to speculate further that the level of free $E_2$ in maternal blood at the time of differentiation of the primitive germ cell could have the same effect as exogenously administered oestrogens, *viz.* to delay or interrupt normal germ cell maturation (Henderson *et al.*, 1983).

Another important descriptive feature of the disease, the rarity of testis cancer in black males (Ross *et al.*, 1979; Newell *et al.*, 1983), also appears compatible with this 'oestrogen excess' hypothesis. During the past several decades, while the incidence of testis cancer has been increasing in white males, the rates in black males have remained stable and are considerably lower. In searching for an explanation for this 'protection' afforded black males, we studied the hormone levels in maternal blood of black and white women during early pregnancy. These women participated in the Collaborative Perinatal Project of the National Institute of Neurological and Communicative Disorders and Stroke (Niswander & Gordon, 1972). In this project more than 55,000 pregnancies were registered at 12 university-affiliated medical centres between 1958 and 1965.

We identified 20 black women who registered in the Project prior to week 12 (measured from the first day of the last menstrual period) of their first pregnancies and who had sera available that had been collected at the time of registration. Each woman's pregnancy proceeded without complication; no woman experienced hyperemesis or toxemia; and all offspring were followed for 7 years with no malformations noted. White women were selected to satisfy the same criteria and each was individually matched to a black woman by medical centre, age (within 1 year), weight (within 4.5 kg) and length of gestation (within 12 days).

Serum samples were stored in a central repository in Bethesda, MD, at −20°C in replicate 4 ml aliquots to prevent unnecessary thawing. The samples of black women were stored, on average, 22 years (standard deviation, s.d. = 1.9 years) and those of white women were stored, on average, 21.8 years (s.d. = 1.8 years). These samples were shipped on dry ice to Endocrine Sciences Laboratory, Tarzana, CA for measurement of $E_2$, sex hormone binding globulin binding capacity (SHBG-bc), testosterone and hCG and to HLJ for measurement of percentage of free (i.e., non-protein bound) $E_2$. $E_2$ was measured by the method of Wu and Lundy (1971); the percentage of free $E_2$ was determined by the equilibrium dialysis method of Pardridge and Mietus (1979); SHBG-bc was measured by the selective ammonium sulfate precipitation technique described in Nankin *et al.* (1975) using a tritiated dihydrotestosterone reference; testosterone was measured by radioimmunoassay as described by Furuyama *et al.* (1970); and hCG was measured by a radioimmunoassay which specifically measures hCG in the presence of human luteinizing hormone (Vaitukaitis *et al.*, 1972). The identity of specimens was not known to the processing laboratories. The only identifier was

*Current address: 8612 Bunnell Drive, Potomac, Maryland, USA.
Correspondence: B.E. Henderson
Received 29 July 1987; and in revised form, 12 November 1987.

a coded number unique for each submission of a specimen. Because samples were collected over a 6 year time frame, we plotted hormone values against the data of sample collection and found no evidence of degradation with increased storage time.

The amount of free $E_2$ was computed as the product of total $E_2$ and the percentage of free $E_2$. Hormone and SHBG-bc values followed a lognormal distribution and logarithmic (base 10) values of these variables were used in all statistical analyses. Statistical analyses were performed using the paired *t*-test, analysis of variance and repeated measures analysis of covariance to adjust for differences in length of gestation, assuming a linear relationship between length of gestation and log hormone measures.

Black and white women were closely matched on age, weight and length of gestation (Table I). Subjects ranged in age from 17 to 26 years. At the start of their pregnancies, subjects ranged in weight from 43 to 79.5 kg. Length of gestation at the time of sample collection ranged from 48 to 80 days since the first day of the last menstrual period. There were no significant differences in length of pregnancy at birth or in the birth weight of offspring.

Black women had testosterone levels that were 48% higher than those of white women during the early weeks of gestation (2-sided, $P = 0.0009$). Black women had total $E_2$ levels that were 37% higher, free $E_2$ levels that were 30% higher and SHBG-bc levels that were 22% higher than those of white women, but none of these results was statistically significant. There were no differences in the level of hCG or the percentage of free $E_2$. Adjustment of these comparisons for length of gestation did not alter the findings. Twelve white women and 11 black women had male offspring, and adjustment for sex of the offspring had no effect on the results presented.

**Table I** Relevant pregnancy characteristics ($\pm$ s.d.) of study subjects and geometric mean hormone levels (with 95% confidence limits) for 20 white and 20 black women registered for their first pregnancies in the Collaborative Perinatal Project. For statistical analyses, paired *t*-tests were used and 2-sided *P* values are presented

| *Variable* | *White women* | *Black women* | *P value* |
|---|---|---|---|
| *Relevant pregnancy characteristics* ($\pm$ *s.d.*) | | | |
| Age (yr) | 20.6 ($\pm$2.5) | 20.6 ($\pm$2.5) | 1.00 |
| Weight (kg) | 56.9 ($\pm$7.4) | 57.3 ($\pm$7.6) | 0.37 |
| Days of gestation at sampling | 66.3 ($\pm$9.2) | 67.4 ($\pm$8.3) | 0.27 |
| Weeks of gestation at birth | 40.3 ($\pm$1.2) | 39.6 ($\pm$1.7) | 0.10 |
| Birth weight of offspring (g) | 3331 ($\pm$353) | 3135 ($\pm$617) | 0.23 |
| *Geometric mean hormone levels* (95% *confidence limits*) | | | |
| Testosterone ($ng\,dl^{-1}$) | 77.3 (65.3, 91.4) | 114.4 (97.3, 134.3) | 0.0009 |
| $E_2$ | | | |
| Total ($pg\,dl^{-1}$) | 138.4 (106.6, 179.5) | 189.4 (136.3, 262.7) | 0.09 |
| Free ($pg\,dl^{-1}$) | 1.28 (1.06, 1.54) | 1.66 (1.25, 2.20) | 0.10 |
| Percent free[a] | 0.96 (0.84, 1.08) | 0.90 (0.79, 1.01) | 0.42 |
| SHBG-bc ($\mu g\,dl^{-1}$) | 4.65 (3.60, 5.99) | 5.69 (4.11, 7.86) | 0.25 |
| hCG ($IU\,ml^{-1}$) | 37.5 (28.8, 48.8) | 37.4 (23.8, 58.9) | 0.99 |

[a]Arithmetic mean.

These findings suggest the possibility that not only $E_2$ levels, but also the amount of testosterone in the circulating maternal blood, are important factors in the development of the testis. Testosterone is necessary for the virilization of the male urogenital tract (Wilson *et al.*, 1981). High maternal testosterone levels such as those observed in black women in this study may ensure this orderly process by crossing the placenta into the foetal circulation. There is evidence that exogenous and endogenous maternal testosterone levels affect foetal genitourinary development. Women treated with certain oral progestins (testosterone analogs) during pregnancy have delivered female offspring with congenital masculinization of the external genitalia suggesting that these steroids have a direct androgenic action on the foetus (Grumbach *et al.*, 1959). The virilizing effects on female foetuses of high levels of endogenous testosterone which were caused by a variety of pregnancy associated ovarian tumours including luteomas and mucinous cystadenomas have been described (Malinak & Miller, 1965; Jenkins *et al.*, 1968; Verhoeven *et al.*, 1973). Thus, the excess of testosterone in the early gestational blood of black women provides a possible explanation for the subsequent lower incidence of testis cancer in black male offspring as it may counteract the effects of elevated oestrogen.

We note that the incidence of cryptorchidism in black males is only one-third that of white males (Heinonen *et al.*, 1977). In rats, oestrogen-inhibited testicular descent can be reversed by treatment with androgens (Rajfer & Walsh, 1977). Furthermore, defects of male sexual differentiation, such as androgen insensitivity (testicular feminisation) syndrome, and gonadal dysgenesis are associated with defects of testosterone action or biosynthesis and males with these conditions show a predisposition to germ cell tumours of the testis (Mishell, 1979; Muller & Skakkebaek, 1984).

We have previously reported that young adult black males have higher levels of circulating testosterone than their white counterparts (Ross *et al.*, 1986). In this paper, we showed that this excess is sufficient to 'explain' the two-fold increased lifetime risk of prostate cancer in blacks compared to whites using a model of 'prostate tissue aging' based on the exponential relationship of cancer risk to exposure time (Pike *et al.*, 1983). It seems reasonable to conjecture that the relative excess of testosterone in early gestational black women predisposes their male offspring to this constitutional development. Unresolved are the reasons for the testosterone excess. As prostate cancer risk and testosterone levels are lower in blacks in West Africa than in U.S. blacks (Ahluwalia *et al.*, 1981), a simple genetic predisposition does not seem to be an appropriate explanation. Malnutrition results in lowered postpubertal testosterone levels in mice (Jean-Faucher *et al.*, 1982) and a reduction in fat intake has been reported to reduce circulating testosterone levels in man (Hill & Wynder, 1979). Thus, a combination of environmental factors such as diet and even specific nutrients, e.g., fat, may be relevant and are worthy of investigation.

In summary, the relative amounts of maternal oestrogen and testosterone, circulating during early gestation when the testis is developing, may play a role in subsequent disease outcomes in male offspring.

This work was supported by grants CA 17054, CA 33512 and CA 00652 from the National Institutes of Health.

**References**

AHLUWALIA, B., JACKSON, M.A., JONES, G.W., WILLIAMS, A.O., RAO, M.S. & RAJGURU, S. (1981). Blood hormone profiles in prostate cancer patients in high-risk and low-risk populations. *Cancer*, **48**, 2267.

BROWN, L.M., POTTERN, L.M. & HOOVER, R.N. (1986). Prenatal and perinatal risk factors for testicular cancer. *Cancer Res.*, **46**, 4812.

BURNS, R.K. (1955). Experimental reversal of sex in the gonads of the opposum didelphis virginiana. *Proc. Natl Acad. Sci.*, **41**, 669.

DEPUE, R.H., PIKE, M.C. & HENDERSON, B.E. (1983). Estrogen exposure during gestation and risk of testicular cancer. *J. Natl Cancer Inst.*, **71**, 1151.

DEPUE, R.H., BERNSTEIN, L., ROSS, R.K., JUDD, H.L. & HENDERSON, B.E. (1987). Hyperemesis gravidarum in relation to estradiol levels, pregnancy outcome and other maternal factors: A seroepidemiologic study. *Am. J. Obstet. Gynecol.*, **156**, 1137.

FURUYAMA, S., MAYES, D.M. & NUGENT, C.A. (1970). A radioimmunoassay for plasma testosterone. *Steroids*, **16**, 415.

GRUMBACH, M.M., DUCHARME, J.R. & MOLOSHOK, R.E. (1959). On the fetal masculinizing action of certain oral progestins. *J. Clin. Endocrinol. Metab.*, **19**, 1369.

HEINONEN, O.P., SLONE, D. & SHAPIRO, S. (1977). Birth defects and drugs in pregnancy. John Wright: Littleton, MA, p. 176.

HENDERSON, B.E., BENTON, B., JING, J., YU, M.C. & PIKE, M.C. (1979). Risk factors for cancer of the testis in young men. *Int. J. Cancer*, **23**, 598.

HENDERSON, B.E., ROSS, R.K., PIKE, M.C. & CASAGRANDE, J.T. (1982). Endogenous hormones as a major factor in human cancer. *Cancer Res.*, **42**, 3232.

HENDERSON, B.E., ROSS, R.K., PIKE, M.C. & DEPUE, R.H. (1983). Epidemiology of testis cancer. In *Urological Cancer*, Skinner, D. (ed) p. 237. Grune & Stratton: New York.

HERBST, A.L., ULFELDER, H. & POSKANZER, D.C. (1971). Adenocarcinoma of the vagina. Association of maternal stilbestrol therapy with tumour appearance in young women. *New Engl. J. Med.*, **284**, 878.

HERBST, A.L., ANDERSON, S., HUBBY, M.M., HAENSZEL, W.M., KAUFMAN, R.H. & NOLLER, K.L. (1986). Risk factors for the development of diethylstilbestrol-associated clear cell adenocarcinoma: A case-control study. *Am. J. Obstet. Gynecol.*, **154**, 814.

HILL, P.B. & WYNDER, E.L. (1979). Effect of a vegetarian diet and dexamethasone on plasma prolactin, testosterone dehydroepiandrosterone in men and women. *Cancer Lett.*, **7**, 273.

JEAN-FAUCHER, C., BERGER, M., DE TURCKHEIM, M., VEYSSIERE, G. & JEAN, C. (1982). Effect of preweaning undernutrition on testicular development in male mice. *Int. J. Andrology*, **5**, 627.

JENKINS, M.E., SURANA, R.B. & RUSSEL-CUTTS, C.M. (1968). Ambiguous genitals in a female infant associated with luteoma of pregnancy. *Am. J. Obstet. Gynecol.*, **101**, 923.

KAUPPILA, A., HUHTANIEMI, I. & YLIKORKALA, O. (1979). Raised serum human chorionic gonadotrophin concentrations in hyperemesis gravidarum. *Br. Med. J.*, **1**, 1670.

MALINAK, L.R. & MILLER, G.V. (1965). Bilateral multicentric ovarian luteomas of pregnancy associated with masculinization of a female infant. *Am. J. Obstet. Gynecol.*, **91**, 251.

McLACHLAN, J.A., NEWBOLD, R.R. & BULLOCK, B. (1975). Reproductive tract lesions in male mice exposed prenatally to diethylstilbestrol. *Science*, **190**, 991.

MISHELL, D.R. (1979). Disorders of sexual differentiation. In *Reproductive endocrinology, infertility and contraception*, Mishell, D.R. Jr. & Davajan, V. (eds) p. 191. F.A. Davis: Philadelphia.

MOSS, A.R., OSMOND, D., BACCHETTI, P., TORTI, F.M. & GURGIN, V. (1986). Hormonal risk factors in testicular cancer: A case-control study. *Am. J. Epidemiol.*, **124**, 39.

MULLER, J. & SKAKKEBAEK, N.E. (1984). Testicular carcinoma *in situ* in children with the androgen insensitivity (testicular feminisation) syndrome. *Br. Med. J.*, **288**, 1419.

NANKIN, H.R., PINTO, R., FAN, D.F. & TROEN, P. (1975). Daytime titers of testosterone, LH, estrone, estradiol and testosterone-binding protein: Acute effects of LH and LH-releasing hormone in men. *J. Clin. Endocrinol. Metab.*, **41**, 271.

NEWELL, G.R., MILLS, P.K. & JOHNSON, D.E. (1984). Epidemiologic comparison of cancer of the testis and Hodgkin's disease among young males. *Cancer*, **54**, 1117.

NISKER, J.A., HAMMOND, G.L., DAVIDSON, B.J. & 4 others (1980). Serum sex hormone binding globulin capacity and the percentage of free estradiol in postmenopausal women with and without endometrial carcinoma. *Am. J. Obstet. Gynecol.*, **138**, 637.

NISWANDER, N.R. & GORDON, M. (1972). *The women and their pregnancies*. Saunders: Philadelphia.

PARDRIDGE, W.M. & MIETUS, L.J. (1979). Transport of steroid hormones through the blood-brain barrier, primary role of albumin-bound hormone. *J. Clin. Invest.*, **64**, 145.

PIKE, M.C., KRAILO, M.D., HENDERSON, B.E., CASAGRANDE, J.T. & HOEL, D.G. (1983). 'Hormonal' risk factors, 'breast tissue age' and the age-incidence of breast cancer. *Nature*, **303**, 767.

RAJFER, J. & WALSH, P.C. (1977). Hormonal regulation of testicular descent: Experimental and clinical observations. *J. Urol.*, **118**, 985.

ROSS, R.K., McCURTIS, J.W., HENDERSON, B.E., MENCK, H.R., MACK, T.M. & MARTIN, S.P. (1979). Descriptive epidemiology of testicular and prostatic cancer in Los Angeles. *Br. J. Cancer*, **39**, 284.

ROSS, R.K., BERNSTEIN, L., JUDD, H.L., HANISCH, R., PIKE, M.C. & HENDERSON, B.E. (1986). Serum testosterone levels in healthy young black and white men. *J. Natl Cancer Inst.*, **76**, 45.

SCHOTTENFELD, D., WARSHAUER, M.E., SHERLOCK, S., ZAUBER, A.G., LEDER, M. & PAYNE, R. (1980). The epidemiology of testicular cancer in young adults. *Am. J. Epidemiol.*, **112**, 232.

SOULES, M.R., HUGHES, C.L., GARCIA, J.A., LIVENGOOD, C.H., PRYSTOWSKY, M.R. & ALEXANDER, E. (1980). Nausea and vomiting of pregnancy: Role of human chorionic gonadotrophin and 17-hydroxyprogesterone. *Obstet. Gynecol.*, **55**, 696.

VAITUKAITIS, J.L., BRAUNSTEIN, G.D. & ROSS, G.T. (1972). A radioimmunoassay which specifically measures human chorionic gonadotrophin in the presence of human luteinizing hormone. *Am. J. Obstet. Gynecol.*, **113**, 751.

VERHOEVEN, A.T.M., MASTBOOM, J.L., VAN LEUSDEN, H.A.I.M. & VAN DER VELDEN, W.H.M. (1973). Virilization in pregnancy coexisting with an (ovarian) mucinous cystadenoma: A case report and review of virilizing ovarian tumors in pregnancy. *Obstet. Gynecol. Survey*, **28**, 597.

WALKER, B.E. (1980). Reproductive tract anomalies in mice after prenatal exposure to DES. *Teratology*, **21**, 313.

WILSON, J.D., GEORGE, F.W. & GRIFFIN, J.E. (1981). The hormonal control of sexual development. *Science*, **211**, 1278.

WU, C. & LUNDY, L. (1971). Radioimmunoassay of plasma estrogens. *Steroids*, **18**, 91.

YASUDA, Y., KIHARA, T. & TANIMURA, T. (1985*a*). Effect of ethinyl estradiol on the differentiation of mouse fetal testis. *Teratology*, **32**, 113.

YASUDA, Y., KIHARA, T., TANIMURA, T. & NISHIMURA, H. (1985*b*). Gonadal dysgenesis induced by prenatal exposure to ethinyl estradiol in mice. *Teratology*, **32**, 219.


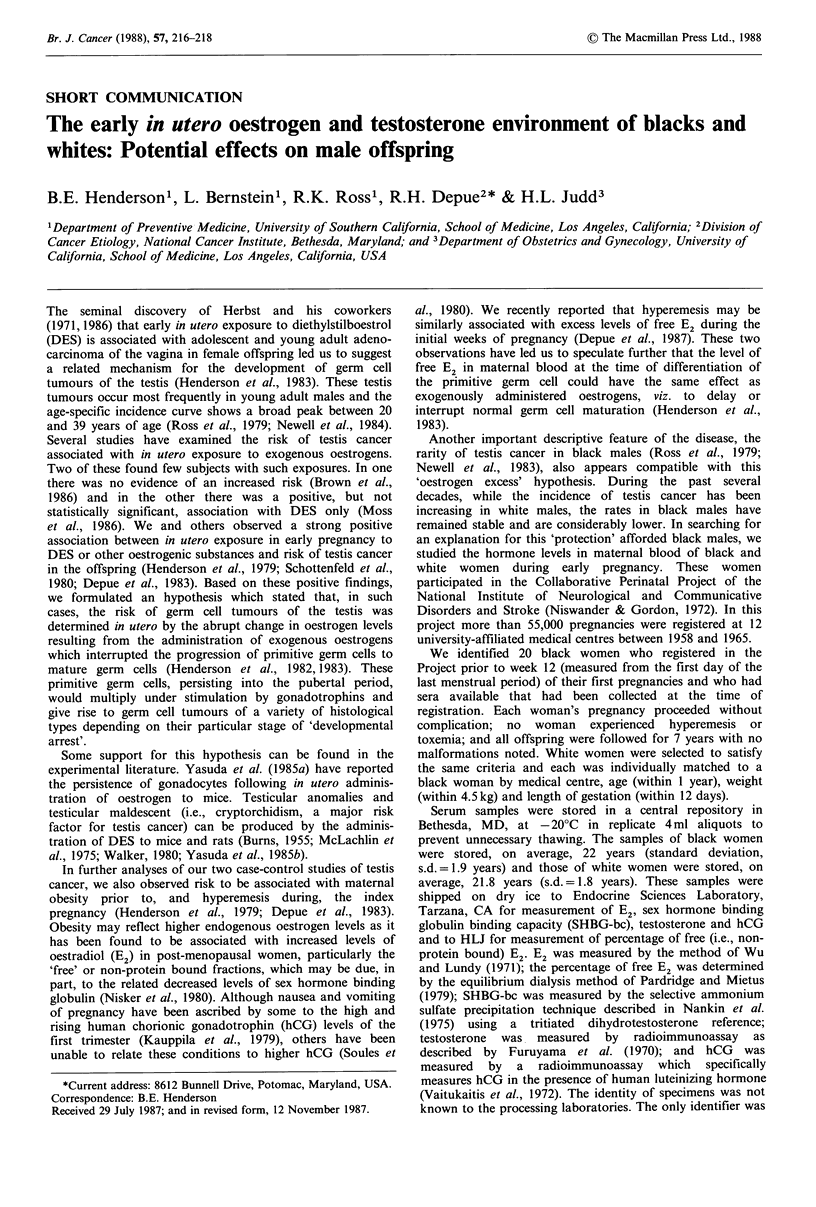

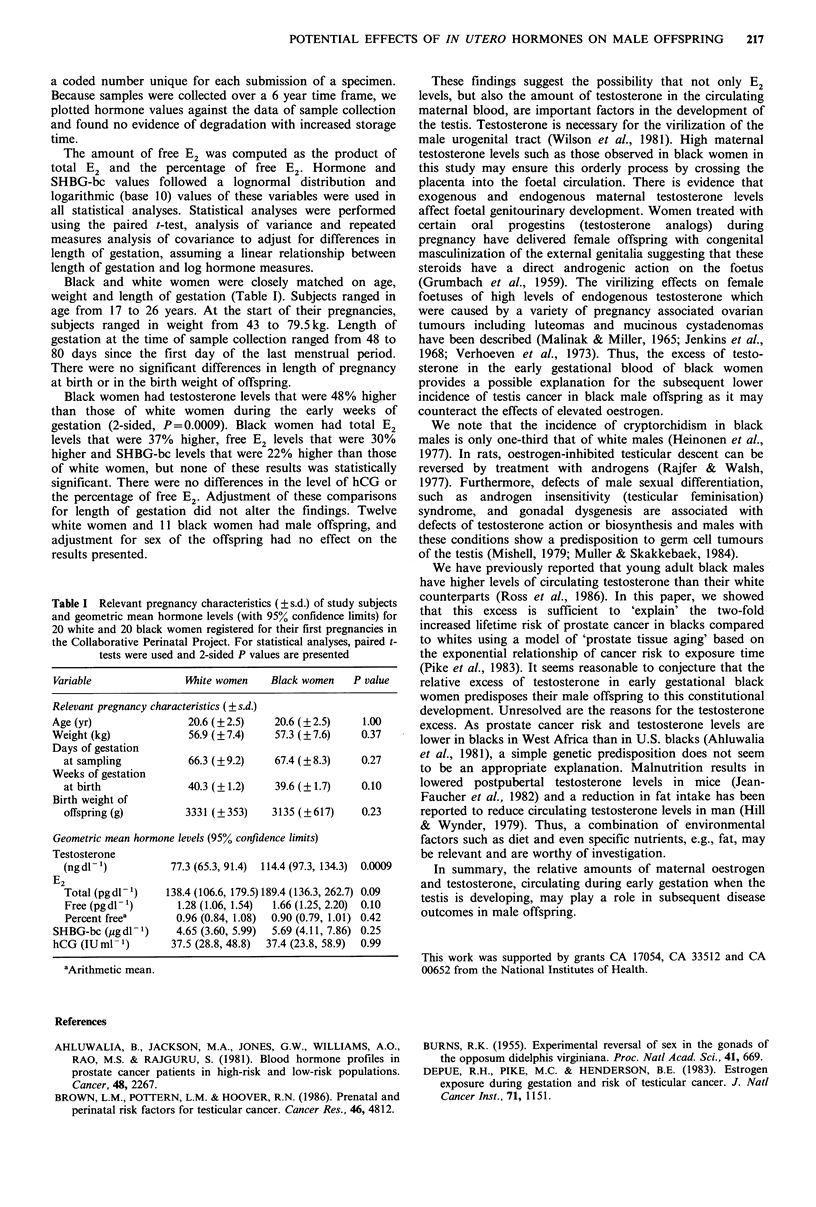

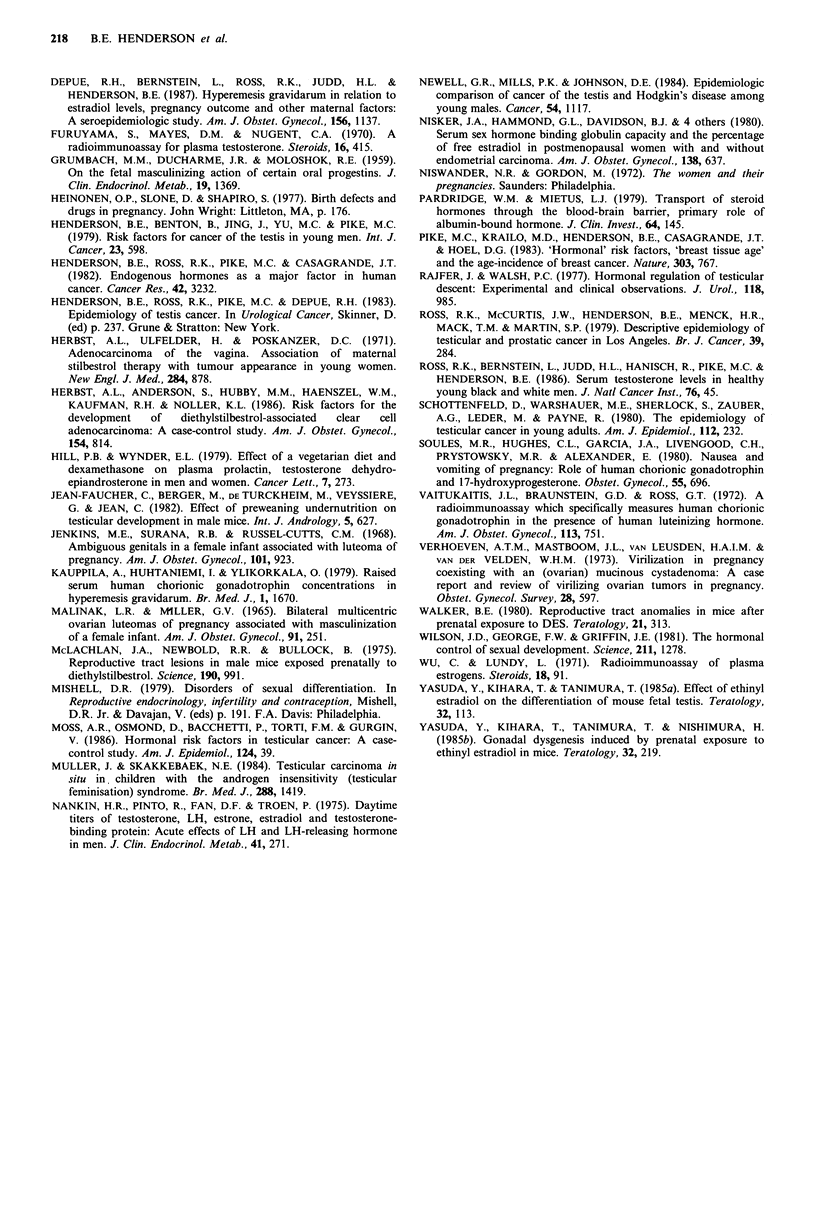


## References

[OCR_00286] Ahluwalia B., Jackson M. A., Jones G. W., Williams A. O., Rao M. S., Rajguru S. (1981). Blood hormone profiles in prostate cancer patients in high-risk and low-risk populations.. Cancer.

[OCR_00292] Brown L. M., Pottern L. M., Hoover R. N. (1986). Prenatal and perinatal risk factors for testicular cancer.. Cancer Res.

[OCR_00296] Burns R. K. (1955). EXPERIMENTAL REVERSAL OF SEX IN THE GON ADS OF THE OPOSSUM DIDELPHIS VIRGINIANA.. Proc Natl Acad Sci U S A.

[OCR_00307] Depue R. H., Bernstein L., Ross R. K., Judd H. L., Henderson B. E. (1987). Hyperemesis gravidarum in relation to estradiol levels, pregnancy outcome, and other maternal factors: a seroepidemiologic study.. Am J Obstet Gynecol.

[OCR_00300] Depue R. H., Pike M. C., Henderson B. E. (1983). Estrogen exposure during gestation and risk of testicular cancer.. J Natl Cancer Inst.

[OCR_00313] Furuyama S., Mayes D. M., Nugent C. A. (1970). A radioimmunoassay for plasma testosterone.. Steroids.

[OCR_00317] GRUMBACH M. M., DUCHARME J. R., MOLOSHOK R. E. (1959). On the fetal masculinizing action of certain oral progestins.. J Clin Endocrinol Metab.

[OCR_00326] Henderson B. E., Benton B., Jing J., Yu M. C., Pike M. C. (1979). Risk factors for cancer of the testis in young men.. Int J Cancer.

[OCR_00331] Henderson B. E., Ross R. K., Pike M. C., Casagrande J. T. (1982). Endogenous hormones as a major factor in human cancer.. Cancer Res.

[OCR_00347] Herbst A. L., Anderson S., Hubby M. M., Haenszel W. M., Kaufman R. H., Noller K. L. (1986). Risk factors for the development of diethylstilbestrol-associated clear cell adenocarcinoma: a case-control study.. Am J Obstet Gynecol.

[OCR_00341] Herbst A. L., Ulfelder H., Poskanzer D. C. (1971). Adenocarcinoma of the vagina. Association of maternal stilbestrol therapy with tumor appearance in young women.. N Engl J Med.

[OCR_00354] Hill P. B., Wynder E. L. (1979). Effect of a vegetarian diet and dexamethasone on plasma prolactin, testosterone and dehydroepiandrosterone in men and women.. Cancer Lett.

[OCR_00359] Jean-Faucher C., Berger M., de Turckheim M., Veyssiere G., Jean C. (1982). Effect of preweaning undernutrition on testicular development in male mice.. Int J Androl.

[OCR_00364] Jenkins M. E., Surana R. B., Russell-Cutts C. M. (1968). Ambiguous genitals in a female infant associated with luteoma of pregnancy. Report of a case.. Am J Obstet Gynecol.

[OCR_00369] Kauppila A., Huhtaniemi I., Ylikorkala O. (1979). Raised serum human chorionic gonadotrophin concentrations in hyperemesis gravidarum.. Br Med J.

[OCR_00374] MALINAK L. R., MILLER G. V. (1965). BILATERAL MULTICENTRIC OVARIAN LUTEOMAS OF PREGNANCY ASSOCIATED WITH MASCULINIZATION OF A FEMALE INFANT.. Am J Obstet Gynecol.

[OCR_00379] McLachlan J. A., Newbold R. R., Bullock B. (1975). Reproductive tract lesions in male mice exposed prenatally to diethylstilbestrol.. Science.

[OCR_00389] Moss A. R., Osmond D., Bacchetti P., Torti F. M., Gurgin V. (1986). Hormonal risk factors in testicular cancer. A case-control study.. Am J Epidemiol.

[OCR_00394] Müller J., Skakkebaek N. E. (1984). Testicular carcinoma in situ in children with the androgen insensitivity (testicular feminisation) syndrome.. Br Med J (Clin Res Ed).

[OCR_00399] Nankin H. R., Pinto R., Fan D. F., Troen P. (1975). Daytime titers of testosterone, LH, estrone, estradiol, and testosterone-binding protein: acute effects of LH and LH-releasing hormone in men.. J Clin Endocrinol Metab.

[OCR_00405] Newell G. R., Mills P. K., Johnson D. E. (1984). Epidemiologic comparison of cancer of the testis and Hodgkin's disease among young males.. Cancer.

[OCR_00410] Nisker J. A., Hammond G. L., Davidson B. J., Frumar A. M., Takaki N. K., Judd H. L., Siiteri P. K. (1980). Serum sex hormone-binding globulin capacity and the percentage of free estradiol in postmenopausal women with and without endometrial carcinoma. A new biochemical basis for the association between obesity and endometrial carcinoma.. Am J Obstet Gynecol.

[OCR_00420] Pardridge W. M., Mietus L. J. (1979). Transport of steroid hormones through the rat blood-brain barrier. Primary role of albumin-bound hormone.. J Clin Invest.

[OCR_00425] Pike M. C., Krailo M. D., Henderson B. E., Casagrande J. T., Hoel D. G. (1983). 'Hormonal' risk factors, 'breast tissue age' and the age-incidence of breast cancer.. Nature.

[OCR_00430] Rajfer J., Walsh P. C. (1977). Hormonal regulation of testicular descent: experimental and clinical observations.. J Urol.

[OCR_00435] Ross R. K., McCurtis J. W., Henderson B. E., Menck H. R., Mack T. M., Martin S. P. (1979). Descriptive epidemiology of testicular and prostatic cancer in Los Angeles.. Br J Cancer.

[OCR_00441] Ross R., Bernstein L., Judd H., Hanisch R., Pike M., Henderson B. (1986). Serum testosterone levels in healthy young black and white men.. J Natl Cancer Inst.

[OCR_00446] Schottenfeld D., Warshauer M. E., Sherlock S., Zauber A. G., Leder M., Payne R. (1980). The epidemiology of testicular cancer in young adults.. Am J Epidemiol.

[OCR_00451] Soules M. R., Hughes C. L., Garcia J. A., Livengood C. H., Prystowsky M. R., Alexander E. (1980). Nausea and vomiting of pregnancy: role of human chorionic gonadotropin and 17-hydroxyprogesterone.. Obstet Gynecol.

[OCR_00457] Vaitukaitis J. L., Braunstein G. D., Ross G. T. (1972). A radioimmunoassay which specifically measures human chorionic gonadotropin in the presence of human luteinizing hormone.. Am J Obstet Gynecol.

[OCR_00463] Verhoeven A. T., Mastboom J. L., van Leusden H. A., van der Velden W. H. (1973). Virilization in pregnancy coexisting with an (ovarian) mucinous cystadenoma: A case report and review of virilizing ovarian tumors in pregnancy.. Obstet Gynecol Surv.

[OCR_00470] Walker B. E. (1980). Reproductive tract anomalies in mice after prenatal exposure to DES.. Teratology.

[OCR_00474] Wilson J. D., George F. W., Griffin J. E. (1981). The hormonal control of sexual development.. Science.

[OCR_00478] Wu C. H., Lundy L. E. (1971). Radioimmunoassay of plasma estrogens.. Steroids.

[OCR_00482] Yasuda Y., Kihara T., Tanimura T. (1985). Effect of ethinyl estradiol on the differentiation of mouse fetal testis.. Teratology.

[OCR_00487] Yasuda Y., Kihara T., Tanimura T., Nishimura H. (1985). Gonadal dysgenesis induced by prenatal exposure to ethinyl estradiol in mice.. Teratology.

